# Docetaxel Oral Delivery System Using Natural Nanoparticles Derived from Ganoderma: Enhanced Pharmacokinetics, Potent Cytotoxicity, and Macrophage-Activating Properties

**DOI:** 10.3390/ph19060899

**Published:** 2026-06-05

**Authors:** Qing Zhao, Ding Ding, Min Zheng, Zhangjin Zheng, Yufeng Yang, Min Lu, Wei Shu, Bingliang Ma

**Affiliations:** 1 Department of Pharmacology, School of Pharmacy, Shanghai University of Traditional Chinese Medicine, Shanghai 201203, China; 15221811720@163.com (Q.Z.); dding0222@163.com (D.D.); zhengminpkyaodong@163.com (M.Z.); zhangjinzheng2025@163.com (Z.Z.); 2 Department of Pharmacy, Jing’an District Zhabei Central Hospital, Shanghai 200070, China; yyf1026548598@163.com; 3 Experiment Center for Science and Technology, Shanghai University of Traditional Chinese Medicine, Shanghai 201203, China; 13122921672@163.com

**Keywords:** natural nanoparticle, docetaxel, oral delivery, Ganoderma, pharmacokinetics, macrophage activation

## Abstract

**Background/Objectives:** Natural macromolecule-based drug delivery carriers have gained extensive attention for biomedical applications. This study aimed to construct an efficient oral delivery system for the widely used antitumor drug docetaxel (DTX) by utilizing natural nanoparticles derived from Ganoderma (LZ-Nnps). Methods: LZ-Nnps loaded with DTX (LZ-Nnps-DTX) were fabricated via an optimized heat-induced self-assembly approach and characterized for morphology, particle size, zeta potential, stability, drug loading, encapsulation efficiency, and molecular interactions with DTX. Intestinal absorption, pharmacokinetics, and tissue distribution were respectively assessed, while antitumor efficacy, macrophage internalization mechanisms, and immunomodulatory activation were further investigated. Results: The optimized formulation showed a particle size of 361.3 ± 5.3 nm, zeta potential of −39.55 ± 1.31 mV, drug loading of 1.51 ± 0.08%, and near-complete encapsulation efficiency (99.97 ± 0.02%)*,* with favorable stability in gastrointestinal fluids. Hydrogen bonding and hydrophobic interactions effectively kept DTX in a stable amorphous state. LZ-Nnps-DTX markedly improved DTX aqueous solubility, dissolution, and intestinal absorption. In vivo assays showed oral LZ-Nnps-DTX achieved 34-fold higher C_max_ and 7.8-fold larger plasma AUC_0-t_ than free DTX, and mainly accumulated in the liver and lung. The nanoparticles entered Caco-2 cells via macropinocytosis and mainly accumulated in the liver. LZ-Nnps-DTX exerted strong cytotoxicity against HepG2, A549, and HCT116 cells, was internalized by RAW264.7 macrophages through caveolae-mediated endocytosis and phagocytosis, and stimulated TNF-α and NO production to suppress tumor growth. Conclusions: These findings demonstrate that LZ-Nnps-DTX effectively enhances oral bioavailability, exerts potent antitumor effects, and activates macrophage-mediated immunity, supporting its promise as an oral DTX delivery system.

## 1. Introduction

Plants and microorganisms are widely used for the synthesis of inorganic nanoparticles, with the remarkable advantages of being simple and environmentally friendly [1,2]. Furthermore, the use of natural macromolecules, such as polysaccharides and proteins, to construct drug delivery systems (DDS) has attracted considerable attention [3]. These natural DDS are biocompatible and efficient [3]. They help enhance drug solubility and dissolution, facilitate intestinal absorption, increase drug concentrations in the systemic circulation, and optimize tissue targeting [3]. Furthermore, natural DDS may synergize with drugs by boosting their efficacy and reducing adverse reactions [3]. Natural nanoparticles (Nnps) formed via the self-assembly of polysaccharides and/or proteins are widely present in the extracts of traditional Chinese medicine (TCM) [4]. These Nnps can act as natural carriers to modify the pharmacokinetic properties of both TCM active constituents [4,5,6] and conventional drugs [7,8]. Notably, Nnps can promote macrophages to release tumor necrosis factor-α (TNF-α) and nitric oxide (NO), indicating immune-enhancing effects [7].

Ganoderma is a well-known TCM prepared from the medicinal mushroom *Ganoderma lucidum* (Leyss. ex Fr.) Karst. or *Ganoderma sinense* Zhao, Xu et Zhang [9]. It is widely used to improve vitality and health in patients [10], especially in those undergoing chemotherapy [11]. Ganoderma contains small-molecule constituents like triterpenoids [12], as well as macromolecules, such as polysaccharides [10] and proteoglycans [13].

Ganoderma polysaccharides are among the most extensively studied TCM-derived polysaccharides [14]. They exhibit anti-inflammatory, immunomodulatory, intestinal flora-regulating, and antitumor effects [14]. Notably, Ganoderma polysaccharides enhance the efficacy of antitumor drugs while reducing their toxicity. For example, they strengthen the pro-apoptotic effect of cisplatin on tongue cancer cells and mitigate cisplatin-induced damage to normal oral epithelial cells [15]. Additionally, oral administration of Ganoderma polysaccharides alleviates gastrointestinal adverse reactions caused by antitumor drugs [16]. Interestingly, these polysaccharides self-assembled into spherical particles, significantly improving their stability and antibacterial activity [17]. It has been reported that Ganoderma polysaccharides form conjugates with methotrexate and further self-assemble into pH-responsive spherical nanoparticles, which improve methotrexate delivery and enhance its antitumor efficacy [18]. Moreover, pH- and glutathione-dual-responsive nanoparticles capable of loading multiple drugs and programmed release have been constructed using Ganoderma polysaccharides [19]. Intestinal cells take up Ganoderma polysaccharides via endocytosis [20]. Ganoderma proteoglycans, glycopeptides, and proteins possess antidiabetic [21], immunomodulatory [22], and anti-inflammatory [23] properties. Following oral administration, Ganoderma proteoglycans are primarily distributed in the intestine, but can also reach the liver [13]. In summary, the aforementioned studies have confirmed that Ganoderma-derived macromolecules exhibit significant biological activity and hold promise as effective delivery materials for chemotherapeutic drugs.

**Figure 1:**
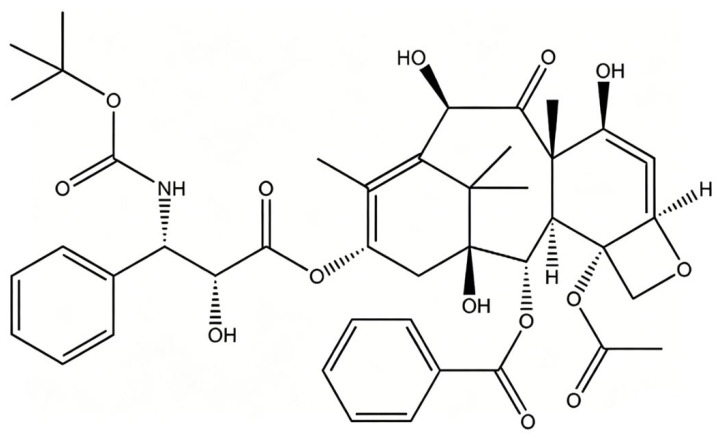
Chemical structure of docetaxel.

Docetaxel (DTX, Figure 1) is a semi-synthetic analog of paclitaxel that is widely used to treat breast and non-small cell lung cancer [24]. However, its clinical administration is primarily via intravenous infusion owing to its low oral bioavailability (<8%) [24]. This poor bioavailability stems from DTX’s low solubility and its first-pass metabolism [24]. Oral DTX administration not only improves patient compliance but also reduces adverse reactions caused by excessive DTX in the circulation. Thus, the development of oral DTX formulations with high bioavailability is of great significance [24]. For example, a hybrid polymeric micelle was developed for the oral delivery of DTX, which increased its oral bioavailability by 10-fold [25]. In 4T1 tumor-bearing BALB/c mice, oral administration of the micelle at a DTX dose of 40 mg/kg yielded antitumor efficacy comparable to that of intravenously injected DTX solution (10 mg/kg), while notably reducing systemic toxicity [25].

Recently, a multicomponent solid self-emulsifying drug delivery system was constructed, where processed *Ganoderma lucidum* spores served as the solid carrier for paclitaxel and *Ganoderma lucidum* spore oil [26]. The developed formulation not only facilitated lymphatic absorption but also strengthened the synergistic immune-mediated antitumor action of paclitaxel [26]. Our previous studies showed that the aqueous extract of Ganoderma forms nanoparticles (size: 381.9 ± 107.0 nm; zeta potential: −26.23 ± 3.16 mV) with DTX and improves DTX pharmacokinetics in mice after oral administration [27]. However, the specific constituents of the Ganoderma extract and their mechanisms of action remain unclear. We hypothesized that Ganoderma-derived macromolecules form Nnps (LZ-Nnps), which can be loaded with DTX to improve its properties. Therefore, this study fabricated DTX-loaded LZ-Nnps (LZ-Nnps-DTX) for oral administration and systematically evaluated (1) their effects on the pharmacokinetic properties of oral DTX, and (2) the cytotoxicity and macrophage activation of LZ-Nnps-DTX themselves.

## 2. Results

### 2.1. Characterization of LZ-Nnps

BCA analysis and phenol-sulfuric acid analysis results revealed that the mass percentage (*w*/*w*, %) of proteins in LZ-Nnps was 106.0 ± 5.8%, while that of polysaccharides was 29.6 ± 1.5%. These results indicated that LZ-Nnps are primarily composed of proteins.

DLS analysis showed that the zeta potential of LZ-Nnps approached approximately 0 at pH 3.0, suggesting that its isoelectric point was approximately 3.0 (Figure S1, Supplementary Materials). Additionally, as the pH increased, the zeta potential shifted from positive to negative, and when the pH exceeded 7, the zeta potential was lower than −23 mV, indicating that LZ-Nnps are less prone to aggregation in alkaline solutions.

Furthermore, DLS analysis demonstrated that when the lyophilized LZ-Nnps powder was dispersed in water, the nanoparticles exhibited a size of 371.9 ± 103.9 nm, a PDI of 0.58 ± 0.07, and a zeta potential of −22.6 ± 0.9 mV.

### 2.2. Characterization of LZ-Nnps-DTX

#### 2.2.1. DLS Analysis

**Figure 2:**
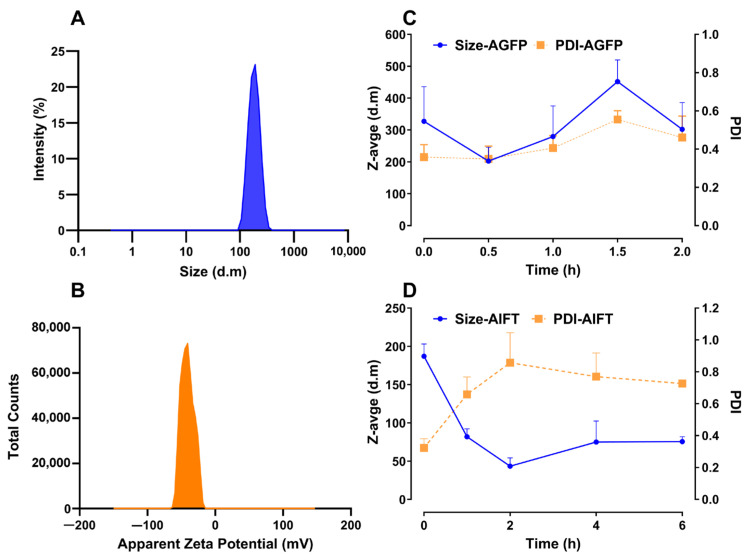
Basic properties of LZ-Nnps-DTX (mean ± SD, n = 3). (**A**) Size, (**B**) Zeta potential, (**C**) Stability in simulated gastric fluid containing pepsin (SGFP), (**D**) Stability in artificial intestinal fluid containing trypsin (SIFT). PDI, polydispersion index.

When the lyophilized LZ-Nnps-DTX powder was dispersed in water, it showed a size of 361.3 ± 5.3 nm (Figure 2A), PDI of 0.48 ± 0.01, and zeta potential of −39.55 ± 1.31 mV (Figure 2B).

#### 2.2.2. Stability in Artificial Gastric and Intestinal Fluids

After incubation in pepsin-containing artificial gastric fluid (Figure 2C), the size of LZ-Nnps-DTX fluctuated between 200 and 500 nm with a slight increase in PDI. Notably, in trypsin-containing artificial intestinal fluid (Figure 2D), the size of LZ-Nnps-DTX decreased significantly within the first 2 h, followed by stabilization until the 6 h incubation endpoint. However, the PDI increased substantially from approximately 0.4 to 0.8 over this period. These results indicate that the nanostructure of LZ-Nnps-DTX may undergo a certain degree of change in the gastrointestinal tract after oral administration, likely due to the effects of digestive enzymes.

#### 2.2.3. SEM, DSC, and PXRD Analysis

**Figure 3:**
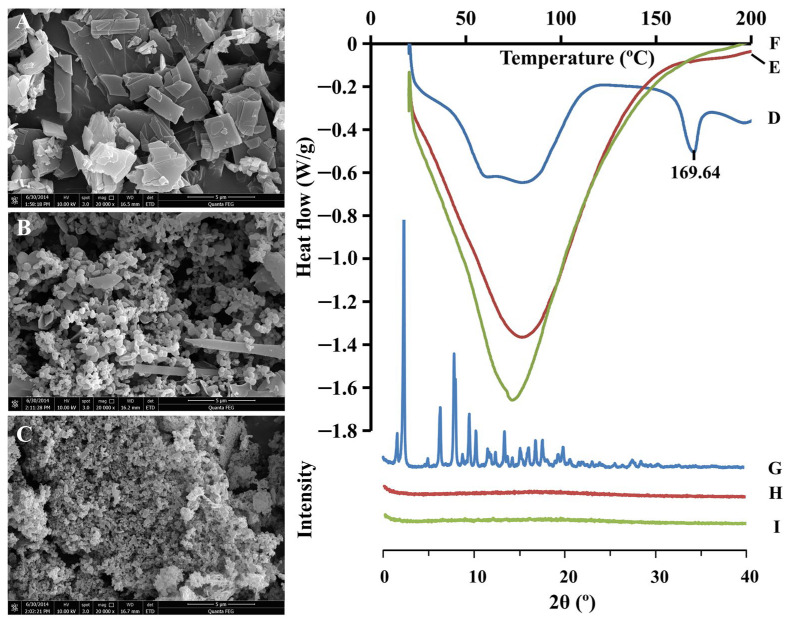
Representative results of scanning electron microscopy ((**A**–**C**); 20,000 ×), differential scanning calorimetry (D–F), and powder X-ray diffraction (G–I) analysis of DTX ((**A**),D,G), LZ-Nnps ((**B**),E,H), and LZ-Nnps-DTX ((**C**),F,I).

SEM images revealed distinct morphologies across the samples (Figure 3A–C). Free DTX exhibited characteristic flake-like crystalline platelets (Figure 3A), empty LZ-Nnps appeared as aggregated granules (Figure 3B), LZ-Nnps-DTX displayed a morphology similar to empty LZ-Nnps, with complete absence of crystalline DTX platelets (Figure 3C), suggesting molecular dispersion of the drug within the nanoparticle matrix. DSC analysis indicated a melting point of 169.64 °C for DTX (Figure 3D); however, no melting points were detected for LZ-Nnps (Figure 3E) or LZ-Nnps-DTX (Figure 3F). The PXRD analysis demonstrated that DTX exhibited numerous strong diffraction peaks (Figure 3G), whereas no diffraction peaks were observed for either LZ-Nnps (Figure 3H) or LZ-Nnps-DTX (Figure 3I). Collectively, these SEM, DSC, and PXRD findings confirm that DTX exists in an amorphous rather than crystalline form within LZ-Nnps-DTX.

#### 2.2.4. FTIR Analysis

**Figure 4:**
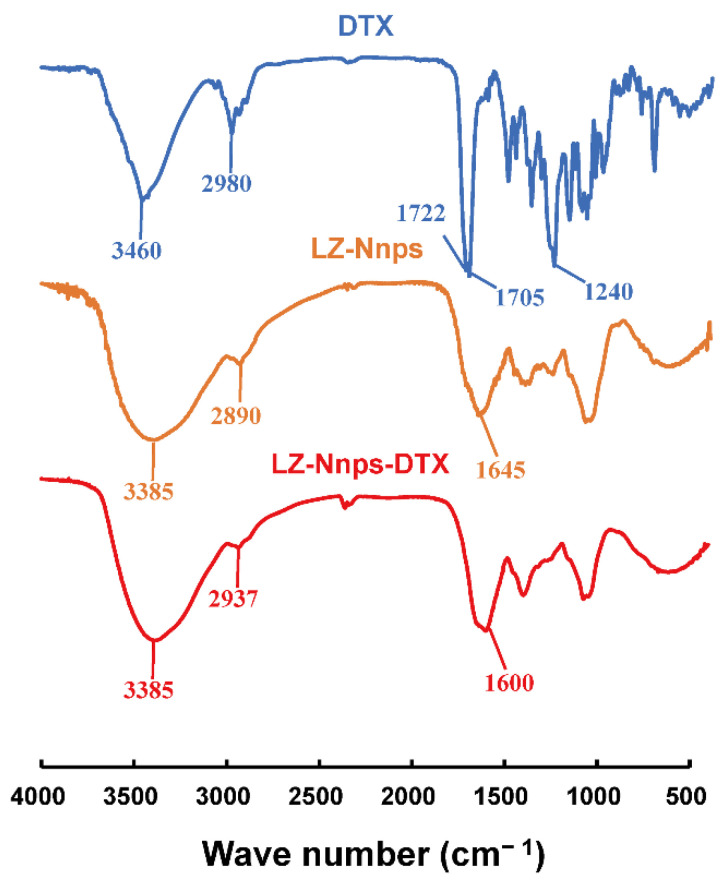
Representative results of Fourier transform infrared spectroscopy analysis of DTX, LZ-Nnps, and LZ-Nnps-DTX.

FTIR spectra (Figure 4) revealed distinct vibrational characteristics for DTX, LZ-Nnps, and LZ-Nnps-DTX. DTX shows O- H stretching at 3460 cm^−1^, C-H stretching at 2980 cm^−1^, and C=O stretching at 1722, 1705, and 1240 cm^−1^, reflecting its hydroxyl, hydrocarbon, and carbonyl groups. LZ-Nnps exhibits broad O-H/N-H stretching at 3385 cm^−1^ (from hydrogen-bonded groups in proteins/polysaccharides), C-H stretching at 2890 cm^−1^, and an amide I band at 1645 cm^−1^ (from peptide bond C=O stretching).

For LZ-Nnps-DTX, the modified C-H stretching at 2937 cm^−1^ (weaker and shifted from both components) indicated hydrophobic interactions. The shift in the amide I band to 1600 cm^−1^ reflects the changes in the LZ-Nnps protein secondary structures due to DTX binding. The carbonyl stretching peaks of DTX at 1722 and 1705 cm^−1^ completely disappeared in the LZ-Nnps-DTX spectrum, indicating that DTX is no longer in its free crystalline state but is molecularly dispersed within the matrix. Overall, DTX interacted with LZ-Nnps via hydrogen bonding and hydrophobic effects, altering the vibrational modes and potentially stabilizing the nanoparticles.

#### 2.2.5. Content, Encapsulation Efficiency, and Drug Loading of DTX

For the LZ-Nnps-DTX formulation, the DTX content was 1.68 ± 0.44%, the drug loading was 1.51 ± 0.08%, and the encapsulation efficiency reached 99.97 ± 0.02%.

### 2.3. Water Solubility and Dissolution of DTX in LZ-Nnps-DTX

**Figure 5:**
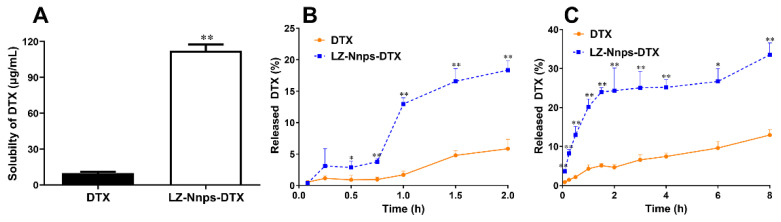
Water solubility (**A**) and dissolution of DTX in simulated gastric fluid (**B**) and in artificial intestinal fluid (**C**) (mean ± SD, n = 3 for solubility; n = 4 for dissolution). *, *p* < 0.05, **, *p* < 0.01 vs. DTX group.

As shown in Figure 5A, the solubility of DTX was 9.8 μg/mL. However, it was significantly enhanced to 112.3 μg/mL, an approximately 11.4-fold increase (*p* < 0.01).

In the SGF (Figure 5B), the dissolution of DTX in the LZ-Nnps-DTX group reached 18.3% at 2 h, whereas that in the DTX group was only approximately 5.8%, with a highly significant difference between the two groups (*p* < 0.01). In SIF (Figure 5C), the dissolution of DTX in the LZ-Nnps-DTX group peaked at 33.5% at 8 h, showing a highly significant increase compared to 12.9% in the DTX group (*p* < 0.01).

### 2.4. Pharmacokinetics of DTX in LZ-Nnps-DTX

#### 2.4.1. In Vitro Absorption in Gut Sacs

**Figure 6:**
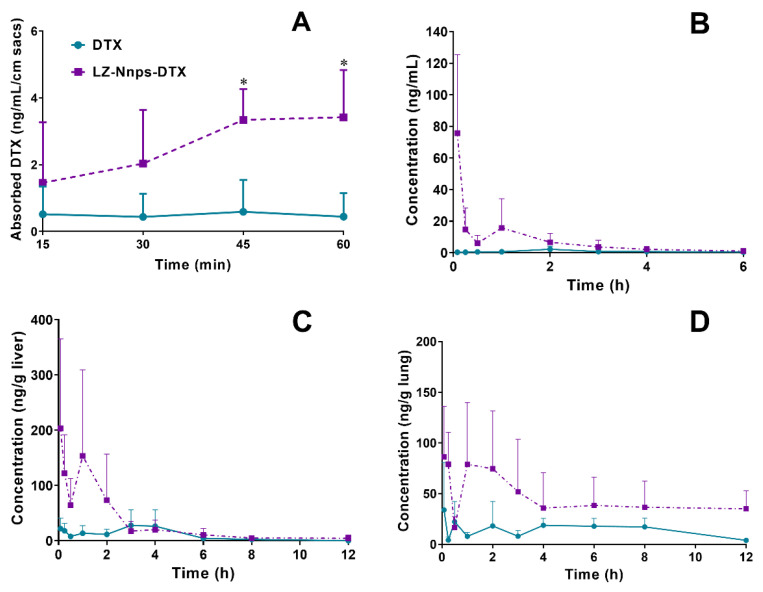
In vitro absorption and in vivo pharmacokinetics of DTX. (**A**) Absorption of free DTX (1 mg/mL) or LZ-Nnps-DTX (at an equivalent DTX dose) in intestinal sacs (mean ± SD, n = 3); (**B**–**D**), Concentration-time curves of DTX in the systemic circulation (**B**), liver (**C**), and lung (**D**) of mice following oral administration of free DTX (20 mg/kg) or LZ-Nnps-DTX (at an equivalent DTX dose) (mean ± SD, n = 5). *, *p* < 0.05 vs. DTX group.

Two-factor ANOVA revealed that the in vitro absorption of DTX in the LZ-Nnps-DTX group was significantly higher than that in the DTX group (Figure 6A, *p* < 0.05). A significant difference was observed after 45 min of incubation (*p* < 0.05).

#### 2.4.2. Pharmacokinetics in Mice

The concentration-time curves of DTX in the systemic circulation, liver, and lungs of mice after oral administration are shown in Figure 6B–D, and the corresponding pharmacokinetic parameters are presented in Table 1.

The results indicated that in the systemic circulation, liver, and lungs, the C_max_ and AUC of DTX were higher in the LZ-Nnps-DTX treatment group than in the DTX group. These results suggest that both the pharmacokinetic profile and distribution of DTX in potential target tissues were improved when administered orally as LZ-Nnps-DTX.

### 2.5. Pharmacokinetics of DiR-Labelled LZ-Nnps-DTX

#### 2.5.1. Labeling of LZ-Nnps-DTX with DiR

As shown in Figure S2 (see Supplementary Materials), the fluorescent dye DiR exhibited a characteristic absorption peak at 748 nm. However, no such peak was observed at 748 nm for the LZ-Nnps-DTX-DiR group. This result suggests that DiR may interact with nanoparticles or become embedded in LZ-Nnps-DTX.

#### 2.5.2. Uptake of LZ-Nnps-DTX-DiR by Caco-2 Cells

**Figure 7:**
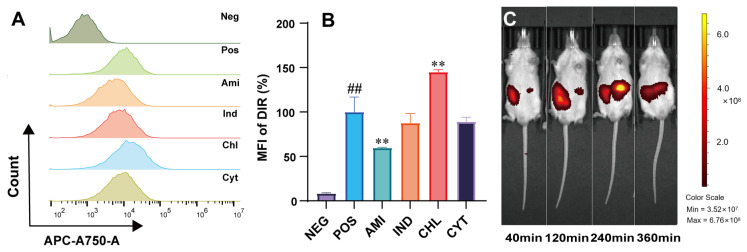
In Vitro uptake and in vivo biodistribution of DiR-labelled LZ-Nnps-DTX. (**A**) Flow cytometry histograms of Caco-2 cells treated with DiR-labelled LZ-Nnps-DTX (100 μg/mL) with or without endocytosis inhibitors (mean ± SD, n = 3); (**B**) Mean fluorescence intensity (MFI) of cells under the same treatment conditions; (**C**) Live imaging of mice after administration of DiR-labelled LZ-Nnps-DTX (100 mg/kg). ^##^, *p* < 0.01 vs. NEG (blank control group); **, *p* < 0.01 vs. POS (positive control group, without endocytosis inhibitors). Inhibitors: AMI (amiloride, 2.5 mM), CHL (chlorpromazine, 10 μg/mL), CYT (cytochalasin D, 5 μM), IND (indomethacin, 100 μg/mL).

As shown in Figure 7A,B, Caco-2 cells exhibited a significantly higher uptake of LZ-Nnps-DTX-DiR than the control group (*p* < 0.01). Additionally, amiloride, a well-characterized macropinocytosis inhibitor, markedly reduced the uptake of LZ-Nnps-DTX-DiR by Caco-2 cells (*p* < 0.01).

#### 2.5.3. Live Imaging Analysis in Mice

After oral administration of an aqueous LZ-Nnps-DTX-DiR solution (100 mg/kg), DiR fluorescence signals initially increased and then decreased (Figure 7C). The liver displayed the strongest signal at 120 min, whereas the abdomen displayed the strongest signal at 240 min. These findings indicate that, following oral administration, LZ-Nnps-DTX-DiR was primarily distributed in the liver and intestine.

### 2.6. In Vitro Bioactivity of LZ-Nnps-DTX

#### 2.6.1. Cytotoxicity of LZ-Nnps-DTX on Tumor Cells

**Figure 8:**
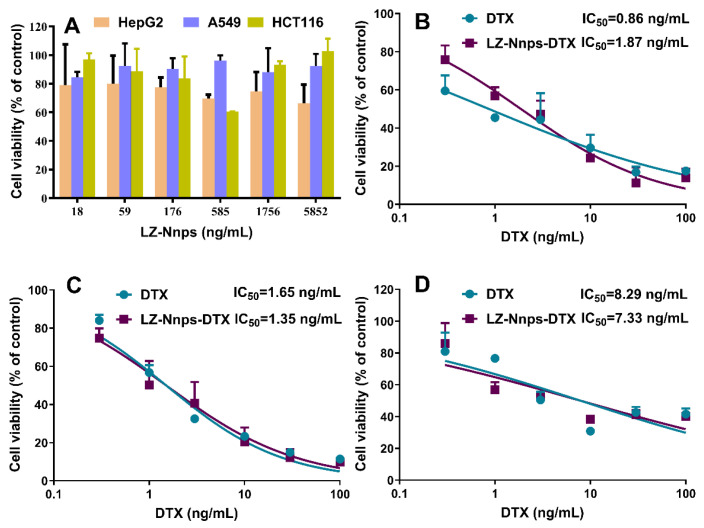
Cytotoxicity of LZ-Nnps (**A**), DTX, and LZ-Nnps-DTX on the cell viability of HepG2 (**B**), A549 (**C**), and HCT116 (**D**) cells (mean ± SD, n = 3).

As shown in Figure 8A, LZ-Nnps alone exhibited weak cytotoxicity against HepG2, A549, and HCT116 cells. Even when the cells were incubated with LZ-Nnps at a concentration of 5852 ng/mL for 48 h, they maintained high viability. In contrast, 48 h incubation with free DTX resulted in a concentration-dependent inhibitory effect on the viability of HepG2, A549, and HCT116 cells, with IC_50_ values of 0.84 ng/mL, 1.65 ng/mL, and 8.29 ng/mL, respectively (Figure 8B–D).

The IC_50_ value of LZ-Nnps-DTX against HepG2 cells was slightly higher than that of free DTX (Figure 8B, *p* < 0.05). However, no significant differences were observed between LZ-Nnps-DTX and free DTX in A549 and HCT116 cells (Figure 8C,D, *p* > 0.05). These results confirmed that DTX retains significant cytotoxicity against tumor cells, even when encapsulated in LZ-Nnps.

#### 2.6.2. Uptake of LZ-Nnps-DTX-DiR by Raw264.7 Cells

**Figure 9:**
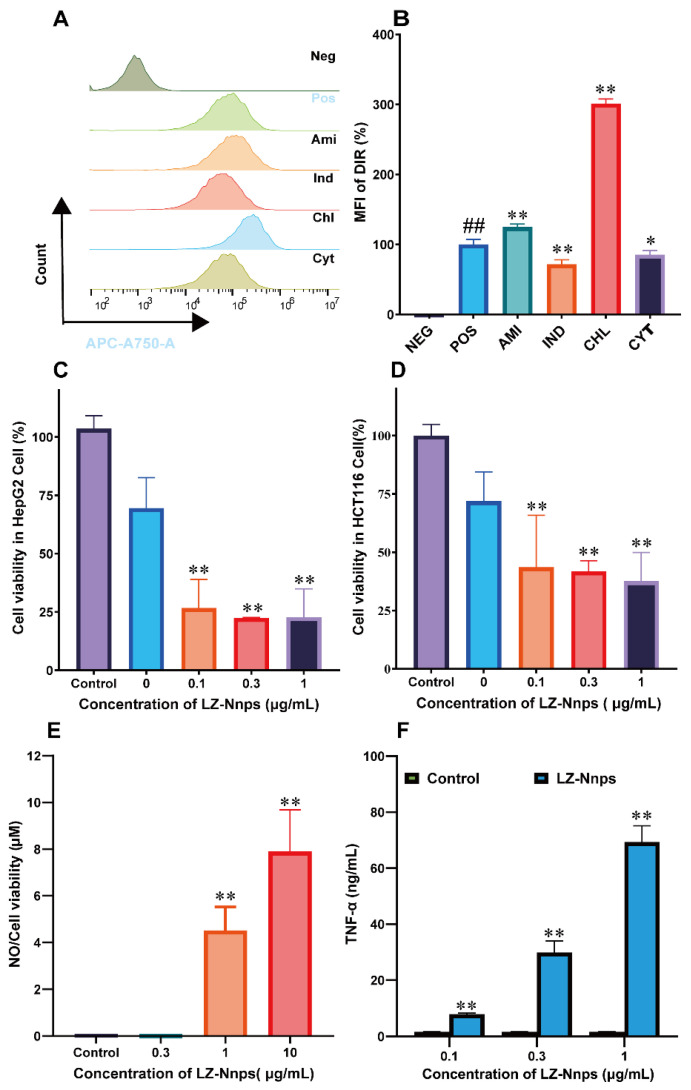
Uptake by and effects on Raw264.7 cells (mean ± SD, n = 3). (**A**) Flow cytometry histograms of Raw264.7 cells treated with DiR-labelled LZ-Nnps-DTX (100 μg/mL) with or without endocytosis inhibitors; (**B**) Mean fluorescence intensity (MFI) of cells under the same treatment conditions; (**C**,**D**), Effects of culture medium from LZ-Nnps-pretreated Raw264.7 cells on viability of HepG2 (**C**) and HCT116 (**D**) cells; E-F, Effects of LZ-Nnps on NO (**E**) and TNF-α (**F**) production in Raw264.7 cells. ##, *p* < 0.01 vs. NEG; *, *p* < 0.05 and **, *p* < 0.01 vs. POS or Control. NEG (blank control group); POS (positive control group, without endocytosis inhibitors). Inhibitors: AMI (amiloride, 2.5 mM), CHL (chlorpromazine, 10 μg/mL), CYT (cytochalasin D, 5 μM), IND (indomethacin, 100 μg/mL).

As shown in Figure 9A,B, Raw264.7 cells took up LZ-Nnps-DTX-DiR. Additionally, indomethacin and cytochalasin D, well-characterized inhibitors of caveolae-mediated and phagocytosis-mediated endocytosis, respectively, exerted strong inhibitory effects (*p* < 0.01) on the uptake of LZ-Nnps-DTX-DiR.

#### 2.6.3. LZ-Nnps Pretreatment Enhanced Macrophage Antitumor Activity

As shown in Figure 9, the culture medium from LZ-Nnps-pretreated macrophages reduced the viability of HepG2 (Figure 9C) and HCT116 cells (Figure 9D), with statistical significance observed in both the cell lines (*p* < 0.01).

#### 2.6.4. LZ-Nnps Stimulate NO and TNF-α Release in Macrophages

LZ-Nnps induced a concentration-dependent increase in the production of NO (Figure 9E) and TNF-α (Figure 9F) in RAW264.7 cells, which underscores its capacity to activate macrophages.

## 3. Discussion

Most studies on Ganoderma macromolecules have focused on polysaccharides; however, this study systematically demonstrated the drug-loading capacity and macrophage activation effects of Ganoderma-derived natural nanoparticles, namely LZ-Nnps.

The LZ-Nnps exhibited a relatively uniform and stable nanostructure and were primarily composed of proteins. A small number of rod-like structures observed in Figure 3B were attributed to residual microfibers (e.g., cellulose) from the Ganoderma aqueous extract; however, these were minor impurities and did not affect the spherical morphology and nanoscale properties of the predominant LZ-Nnps population. Polysaccharides typically have high molecular weights and large sizes [14], and are therefore easy to remove during centrifugation and filtration. Notably, the total measured protein and polysaccharide contents in LZ-Nnps exceeded 100%. This discrepancy may stem from carbohydrate interference in protein quantification [28]. In addition, it is important to note that the protein and polysaccharide levels in LZ-Nnps were quantified using BSA and glucose as reference standards, respectively. These values represent the relative amounts of the two constituents of the LZ-Nnps. It is generally accepted that during heating, polysaccharides may react with proteins to form complexes [29]; such complexes help enhance the stability of self-assembled nanoparticles and modulate the release profiles of loaded drugs [30]. However, the specific role of the small amount of polysaccharides present in LZ-Nnps-DTX remains to be elucidated. Additionally, detailed structural analysis of the protein and polysaccharide components will be conducted in our follow-up studies.

Common strategies for constructing self-assembled protein nanoparticles include pH-, urea-, alcohol-, high pressure-, and heat-mediated dissociation and recombination [31]. Based on our previous studies, this study adopted a heat-induced strategy, which is consistent with the traditional heating-based extraction methods for TCM. Orthogonal experiments were conducted to investigate the effects of heating temperature, carrier concentration, drug-to-carrier ratio, and solution pH on the properties of LZ-Nnps-DTX, including particle size, PDI, zeta potential, DTX loading capacity, and DTX encapsulation efficiency. Based on these results, the optimal preparation protocol for LZ-Nnps-DTX was adopted.

The zeta potential of LZ-Nnps-DTX was as low as −39.55 mV, indicating a low tendency for aggregation [32]. The good stability of LZ-Nnps-DTX in artificial gastric fluid and intestinal fluid was consistent with this result. DTX’s encapsulation efficiency exceeded 99%, suggesting that it was nearly fully encapsulated or tightly adsorbed by the LZ-Nnps. Nevertheless, LZ-Nnps-DTX exhibited a relatively large particle size and PDI, reflecting suboptimal uniformity, and LZ-Nnps had a comparatively low drug-loading capacity. These characteristics are common limitations of naturally derived macromolecular nanoparticles [33]. However, these drawbacks are considered acceptable for oral drug formulation. In this study, LZ-Nnps-DTX was formulated into powder and stored under refrigeration. The powder was reconstituted into solution immediately prior to use, which helped maintain the quality of LZ-Nnps-DTX. Nevertheless, the storage stability of the LZ-Nnps-DTX powder remains to be further investigated.

Solubility and dissolution are two key factors that influence the absorption of oral drugs. In this study, loading DTX into LZ-Nnps increased its solubility by approximately 11.4-fold while significantly enhancing DTX dissolution in both simulated gastric and intestinal fluids. SEM, DSC, and PXRD results confirmed that DTX existed in an amorphous state within LZ-Nnps-DTX. Notably, although few irregular fragments were observed in the SEM image of LZ-Nnps-DTX (Figure 3C), these were attributed to lyophilization-induced aggregation or collapse of the protein matrix rather than crystalline DTX, as corroborated by the absence of melting points in DSC (Figure 3F) and diffraction peaks in PXRD (Figure 3I). The results of FTIR analysis indicate that there are multiple interactions between DTX and LZ-Nnps, which are conducive to inhibiting the formation of DTX crystals. Drugs in the amorphous form typically exhibit better solubility and dissolution than their crystalline counterparts [34], explaining the improved solubility and dissolution of DTX when formulated as LZ-Nnps-DTX.

In the dissolution study, DTX exhibited slow release in the initial phase, followed by significantly faster release in the later phase. The initial release likely originated primarily from DTX adsorbed on the surface of the LZ-Nnps. Given the high encapsulation efficiency of DTX, these dissolution results indicate that most DTX is embedded within the nanoparticles rather than being adsorbed on their surfaces. As the incubation time increased, the embedded DTX was rapidly released. This accelerated release is likely attributable to conformational changes in LZ-Nnps induced by simulated gastric and intestinal fluids, an observation supported by variations in LZ-Nnps’ particle size and PDI of the LZ-Nnps.

Pharmacokinetic studies showed that compared with the control group, the LZ-Nnps-DTX group had significantly higher DTX exposure levels (C_max_ and AUC_0-t_) in the systemic circulation, liver, and lungs of mice. The intestinal sac experiments explained this improvement, mainly because of the improved intestinal absorption of DTX in the LZ-Nnps-DTX group. This increase in intestinal absorption is related to the enhanced solubility and dissolution of DTX; however, other mechanisms also play a role. For example, fluorescein DiR-labelled LZ-Nnps-DTX can be taken up by Caco-2 cells via macropinocytosis, a process that helps reduce DTX metabolism mediated by CYP3A4 [35] and efflux mediated by P-glycoprotein [36], thereby further enhancing its absorption. Additionally, live imaging studies revealed that LZ-Nnps-DTX is absorbed and distributed to the liver. As a result, the higher DTX exposure in the LZ-Nnps-DTX group may be due to the nanoparticles being absorbed into the circulation, not just free DTX. However, the detailed in vivo process of LZ-Nnps-DTX requires clarification.

The results of the CCK-8 experiments demonstrated that LZ-Nnps did not substantially decrease the vitality of the tumor cells following a 48 h incubation period, suggesting that LZ-Nnps did not directly inhibit tumor cell growth. It is generally anticipated that the cytotoxic effect of a drug will be greatly diminished if its size dramatically increases following nanoparticle creation, as this is not favorable for drug entry into cells. The present study indicates that DTX in LZ-Nnps-DTX may be readily absorbed and released by cancer cells, as evidenced by the fact that the inhibitory effects on HepG2 cells were only marginally diminished, while on A549 and HCT116 cells, the effects were largely preserved.

Plant-derived proteins and polysaccharides are widely recognized for their immunomodulatory effects [14]. Tumor-associated macrophages, which include M1 and M2 subtypes, play critical roles in cancer progression [37]. In general, M1 macrophages exert antitumor effects by expressing high levels of inducible nitric oxide synthase and TNF-α, whereas M2 macrophages exhibit pro-tumor properties [37]. Therefore, activation of macrophages to release TNF-α and NO can enhance their antitumor activity. In this study, LZ-Nnps were taken up by RAW264.7, and subsequently stimulated the secretion of NO and TNF-α, thereby inhibiting the viability of tumor cells. These findings suggest that when LZ-Nnps are used to deliver DTX, they not only improve DTX absorption and systemic exposure but also produce synergistic antitumor efficacy through macrophage activation. Interestingly, chlorpromazine enhanced LZ-Nnps-DTX-DiR uptake. This phenomenon has also been documented in research on other self-assembled nanoparticles, although the exact underlying mechanism remains unclear [7,8].

It is noteworthy that LZ-Nnps-DTX exhibits several favorable characteristics distinct from those of reported nanoparticle-based oral DTX delivery systems. First, the carrier is derived from plant components with a long history of clinical use, offering favorable biocompatibility and safety profiles while avoiding the extensive use of organic reagents commonly required in the preparation of synthetic nano-delivery systems. Second, LZ-Nnps-DTX substantially enhances the intestinal absorption and pharmacokinetic profiles of DTX, as reflected by a 34-fold increase in C_max_ and a 7.8-fold increase in AUC_0-t_. These improvements appear considerably more pronounced than those reported for certain existing formulations, such as a polymeric micelle that increased the C_max_ of DTX (10 mg/kg) from 158 ng/mL to 222.3 ng/mL [25]. Third, LZ-Nnps-DTX achieves an encapsulation efficiency of 99.97%, which compares favorably with most reported DTX nanoformulations, including the polymeric micelle with a reported encapsulation efficiency of 33.3% [25]. Finally, while conventional synthetic carriers typically serve primarily as passive drug containers, LZ-Nnps intrinsically activate macrophages, thereby providing a dual chemo-immunotherapeutic mechanism not typically observed with traditional polymeric or lipid-based platforms.

## 4. Materials and Methods

### 4.1. Materials

Vitamin C was purchased from TCI (Shanghai) Development Co., Ltd. (Shanghai, China). Amiloride, carbamazepine, dialysis bags (3500 Da), and filters (0.22 μm) were obtained from Shanghai Yuanye Biological Co., Ltd. (Shanghai, China). CCK-8 kits, cytochalasin D, 1, 1′-dioctadecyl-3, 3, 3’, 3′-tetramethylindotricarbocyanine iodide (DiR), docetaxel, and indomethacin were purchased from Dalian Meilun Biotechnology Co., Ltd. (Dalian, China). The bicinchoninic acid (BCA) protein assay kit was provided by Shanghai Biyuntian Biotechnology Co., Ltd. (Shanghai, China). The TNF-α assay kit was purchased from Lianke Biotechnology Co., Ltd. (Wuhan, China). Pepsin was obtained from NeoFroxx GmbH (Einhausen, Germany). Ammonium formate, formic acid, trypsin, and Dulbecco’s Modified Eagle Medium (DMEM) were purchased from Thermo Fisher Scientific (Waltham, MA, USA). Fetal bovine serum (FBS) and a penicillin–streptomycin solution were purchased from Biological Industries (BioInd) (Beit-Haemek, Israel). Zoletil^®^ 50 (an injection containing tiletamine hydrochloride and zolazepam hydrochloride) was purchased from Virbac Trading (Shanghai) Co., Ltd. (Shanghai, China). Dimethyl sulfoxide (DMSO), chlorpromazine, and N-(1-naphthalene) ethylenediamine dihydrochloride were purchased from Merck (Darmstadt, Germany). Methanol was purchased from Honeywell Trading (Shanghai) Co., Ltd. (Shanghai, China). The purity of all reference compounds used in this study exceeded 98%.

Dried Ganoderma herbal pieces (Batch Nos. 220812 and 231220) were prepared from *Ganoderma lucidum* (Leyss. ex Fr.) Karst was purchased from Shanghai Kang Qiao Herbal Pieces Co., Ltd. (Shanghai, China).

### 4.2. Extraction, Isolation, and Quality Control of LZ-Nnps

First, an aqueous extract of Ganoderma was prepared according to our previously reported method [27]. Briefly, Ganoderma herbal pieces were crushed, soaked in 10 volumes of water for 0.5 h, and extracted twice with boiling water (1.5 h for the first extraction and 1 h for the second). The aqueous extract was vacuum-dried at 60 °C.

Next, the LZ-Nnps powder was obtained by centrifugation, filtration, dialysis, and lyophilization. Briefly, the Ganoderma extract was dissolved in water to a concentration of 0.15 g/mL. The solution was thoroughly stirred, sonicated for 1.5 h, and then centrifuged at 3000 rpm for 10 min. The resulting supernatant was filtered through a 0.22 μm microporous membrane, and the filtrate was dialyzed (molecular weight cutoff: 3500 Da) for 3 days. Finally, the residue in the dialysis bag was collected and lyophilized at −45 °C to yield the LZ-Nnps powder.

For quality control, the LZ-Nnps powder was dissolved in water to a concentration of 1 mg/mL and sonicated for 1.5 h. Protein concentration was determined using a BCA assay, while polysaccharide concentration was measured using the phenol–sulfuric acid method.

### 4.3. Preparation of LZ-Nnps-DTX

LZ-Nnps-DTX was prepared from LZ-Nnps using an optimized heat-induced self-assembly method. The key factors influencing the properties of the resulting LZ-Nnps-DTX include the LZ-Nnps concentration, DTX-to-LZ-Nnps concentration ratio, solution pH, and heating temperature. Therefore, an orthogonal experiment was conducted to determine the optimal preparation conditions (see Supplementary Materials).

LZ-Nnps-DTX was then prepared following the optimized procedure outlined below: (1) Disperse LZ-Nnps powder in water to a concentration of about 5 mg/mL; (2) Heat the LZ-Nnps aqueous solution to 70 °C and maintain this temperature for 0.5 h; (3) Remove undispersed material via centrifugation at 10,000 rpm for 10 min; (4) Quantify Ganoderma protein content in the resulting supernatant using a BCA assay, then dilute the supernatant to a final Ganoderma protein concentration of 1 mg/mL; (5) While stirring (500 rpm), add a DTX ethanol solution to the LZ-Nnps solution, then adjust the mixture to achieve a final DTX concentration of 0.3 mg/mL, with ethanol content kept below 1%; (6) Adjust the solution pH to 10 and continue stirring at 500 rpm for 12 h; (7) Centrifuge the solution at 10,000 rpm for 10 min, then lyophilize the supernatant to obtain LZ-Nnps-DTX powder.

### 4.4. Characterization of LZ-Nnps-DTX

#### 4.4.1. Dynamic Light Scattering (DLS) Analysis

Lyophilized powders of LZ-Nnps or LZ-Nnps-DTX were dispersed in water (1 mg/mL) and vortexed thoroughly. After centrifugation at 10,000 rpm for 10 min, DLS analysis was performed to determine the particle size, polydispersity index (PDI), and zeta potential of the nanoparticles in the supernatant using a Malvern Zetasizer Nano Analyzer (Worcestershire, UK).

#### 4.4.2. Stability of LZ-Nnps-DTX in Simulated Gastric and Intestinal Fluids

Lyophilized LZ-Nnps-DTX powder was dissolved in simulated gastric fluid [SGFP: aqueous solution of sodium chloride (2.0 g/L) containing 0.32% (*w*/*v*) pepsin, pH 1.2] or simulated intestinal fluid [SIFT: aqueous solution of sodium phosphate (0.2 mol/L) containing 1% (*w*/*v*) trypsin, pH 6.8] to a final concentration of 1 mg/mL. The solutions were incubated at 37 °C for predetermined times: 0, 30, 60, 90, and 120 min for SGFP and 0, 1, 2, 4, and 6 h for SIFT. At each time point, the volume of the supernatant was sampled, and the particle size and PDI of LZ-Nnps-DTX were immediately measured using DLS.

#### 4.4.3. Scanning Electron Microscopy (SEM) Analysis

DTX, LZ-Nnps, and LZ-Nnps-DTX powders were sputter-coated with gold and vacuum-dried. Their morphological characteristics were observed using a FEI Quanta 250 scanning electron microscope (FEI Company, Hillsboro, OR, USA) operated at 10 kV.

#### 4.4.4. Differential Scanning Calorimetry (DSC) Analysis

DSC analysis was performed using a TA DSC Q2000 differential scanning calorimeter (TA Instruments, New Castle, DE, USA). Briefly, DTX, LZ-Nnps, and LZ-Nnps-DTX powders were placed in open aluminum crucibles and heated from 20 to 320 °C at a heating rate of 10 °C/min.

#### 4.4.5. Powder X-Ray Diffraction (PXRD) Analysis

PXRD analysis of the DTX, LZ-Nnps, and LZ-Nnps-DTX powders was conducted using a Bruker D2 Phaser system (Rheinstetten, Germany). The operating conditions were as follows: voltage 30.0 kV, current 10.0 mA, scanning range 3–40°, step size 0.02°, and scanning speed 0.1 s per step.

#### 4.4.6. Fourier Transform Infrared Spectroscopy (FTIR) Analysis

Briefly, 10.0 mg of each powder (DTX, LZ-Nnps, or LZ-Nnps-DTX) was ground into a fine powder with approximately 190 mg of potassium bromide. Each mixture was then pressed into pellets. FTIR spectra of the samples were acquired using an IRAffinity-1S FTIR spectrometer (SHIMADZU, Kyoto, Japan) at a resolution of 4 cm^−1^ and over a wavenumber range of 4000–500 cm^−1^.

#### 4.4.7. Liquid Chromatography-Tandem Mass Spectrometry (LC-MS/MS) for DTX

DTX concentration was quantified using the LC-MS/MS method described in our studies [27]. Briefly, the samples were precipitated with three volumes of acetonitrile and analyzed using an LC-MS/MS system comprising a Waters ACQUITY ultra-high-performance liquid chromatography (UPLC) system (Waters Corporation, Milford, MA, USA) and an API 5500 mass spectrometer (AB Sciex LLC, Framingham, MA, USA) equipped with an electrospray ionization source. Separation was performed on an ACQUITY BEH C18 column (2.1 × 100 mm, 1.7 μm; Waters) using a gradient elution. DTX (*m*/*z* 808.4→527.4) and the internal standard carbamazepine (*m*/*z* 237.0→193.4) were detected via multiple reaction monitoring in the positive ion mode. The method exhibited good linearity over a concentration range of 0.39–800.0 ng/mL and met the requirements for the quantitative analysis of DTX.

#### 4.4.8. Content, Encapsulation Efficiency, and Drug Loading of DTX in LZ-Nnps-DTX

Lyophilized LZ-Nnps-DTX powder was accurately weighed, and the mass was recorded as the M_otal_. A 4 mL aqueous LZ-Nnps-DTX solution was then prepared at a concentration of 1 mg/mL.

Approximately 2 mL of the LZ-Nnps-DTX aqueous solution was mixed with 8 mL of methanol and sonicated for 30 min. The mixture was centrifuged at 10,000 rpm for 10 min, and the concentration of DTX in the supernatant was measured using a previously described LC-MS/MS method. M_total DTX_ (total DTX in the LZ-Nnps-DTX powder) was calculated by multiplying the detected DTX concentration by the total solution volume. The percentage content of DTX is calculated based on the ratio of M_total DTX_ to M_total_.

About 0.5 mL of the LZ-Nnps-DTX aqueous solution was transferred to an ultrafiltration centrifuge tube (molecular weight cutoff: 3000 Da). After centrifugation at 4200 rpm for 20 min, the filtrate was collected, and the concentration of free DTX was measured via LC-MS/MS. M_free DTX_ (free DTX in the LZ-Nnps-DTX powder) was calculated using the detected free DTX concentration and total solution volume.

The encapsulation efficiency and drug loading capacity of DTX in LZ-Nnps-DTX were calculated using Equations (1) and (2), respectively:

(1) $$\mathrm{Encapsulation}\;\mathrm{efficiency}\;=\;\frac{M_{\mathrm{total}\;\mathrm{DTX}}-M_{\mathrm{free}\;\mathrm{DTX}}}{M_{\mathrm{total}\;\mathrm{DTX}}}\times 100\%$$

(2) $$\mathrm{Drug}\;\mathrm{loading}=\frac{M_{\mathrm{total}\;\mathrm{DTX}}-M_{\mathrm{free}\;\mathrm{DTX}}}{M_{\mathrm{total}}-M_{\mathrm{total}\;\mathrm{DTX}}}\times 100\%$$

### 4.5. Saturated Water Solubility of DTX

First, supersaturated aqueous solutions of DTX and LZ-Nnps-DTX powders were prepared, each containing 1 mg/mL DTX, which far exceeded the reported aqueous solubility of DTX (7.0 μg/mL) [7,8]. The solution was sonicated for 2 h and centrifuged at 14,000 rpm for 10 min. The supernatant was diluted with a 50% (*v*/*v*) methanol–water solution containing the internal standard carbamazepine, and DTX concentrations were determined using a previously described LC-MS/MS method.

### 4.6. Dissolution of DTX

Accurately weighed lyophilized powders of DTX or LZ-Nnps-DTX were added to either simulated gastric fluid [SGF: 0.2% (*w*/*v*) sodium chloride with 0.7% (*v*/*v*) concentrated HCl, pH 1.2] or simulated intestinal fluid [SIF: 680.5 mg potassium dihydrogen phosphate and 89.6 mg sodium hydroxide in 100 mL water, pH 6.8]. The final DTX concentration in all buffers was standardized to 0.2 mg/mL.

Approximately 1 mL of each buffer containing the test substance was transferred to a dialysis bag (molecular weight cutoff: 3500 Da), which was then sealed. The dialysis bag was immersed in a beaker containing 100 mL of the corresponding simulated fluid (SGF or SIF). The beaker was then placed in a water bath at 37 °C with constant stirring at 50 rpm. For the SGF experiments, 800 μL aliquots were sampled at 5, 15, 30, 45, 60, 90, and 120 min. For the SIF experiments, 800 μL aliquots were sampled at 5, 15, and 30 min, and 1, 1.5, 2, 3, 4, 6, and 8 h. After each sampling, 800 μL of blank buffer was immediately added to maintain volume consistency. The DTX concentrations in the samples were determined using a previously described LC-MS/MS method.

### 4.7. Pharmacokinetics of DTX in LZ-Nnps-DTX

#### 4.7.1. In Vitro Absorption in Gut Sacs

All animal experiments were conducted in compliance with the ARRIVE guidelines, the U.K. Animals (Scientific Procedures) Act 1986 and its associated guidelines, EU Directive 2010/63/EU for animal experiments, and the National Institutes of Health (NIH) Guide for the Care and Use of Laboratory Animals (Publication No. 8023, revised 1978). The study was approved by the Institutional Animal Care and Use Committee of Shanghai University of Traditional Chinese Medicine (approval number: PZSHUTCM2404280008) and conducted in accordance with the guidelines of the committee.

After the mice were euthanized by cervical dislocation, the mouse ileal segments were used to compare the in vitro absorption of free DTX and DTX encapsulated in LZ-Nnps-DTX. First, one end of each ileal segment was ligated. The mucosal side of the sac was then filled with Krebs–Ringer buffer containing either free DTX or LZ-Nnps-DTX, with DTX concentrations standardized to 1 mg/mL for both groups. After tightly ligating the other end, the intestinal sac was immediately incubated in 20 mL of oxygenated Krebs–Ringer buffer (37 °C) in a Magnus bath. At 15, 30, 45, and 60 min post-incubation, 200 μL aliquots of bath buffer were sampled, and an equal volume of blank Krebs–Ringer buffer was promptly replenished.

The DTX concentrations in the samples were determined using a previously described LC-MS/MS method. The absorption values were normalized to the length of each intestinal sac, which was measured at the end of the experiment.

#### 4.7.2. Pharmacokinetics of DTX in Mice

One hundred specific-pathogen-free (SPF) male Kunming mice (body weight: 22–24 g) were purchased from the Beijing Vital River Laboratory Animal Technology Co., Ltd. (Beijing, China). Mice were housed in an air-conditioned room maintained at 22–24 °C with a 12 h light/12 h dark cycle. Prior to the experiment, mice were fasted for 12 h with free access to water.

Mice were randomly divided by body weight into two main groups, each subdivided into 10 subgroups. One main group (i.e., 10 subgroups) received an oral aqueous solution of free DTX at a dose of 20 mg/kg (consistent with previous studies using mice [7,8]), whereas the other group received an oral aqueous solution of LZ-Nnps-DTX containing an equivalent DTX dose. At each time point post-administration (0.083, 0.25, 0.5, 1, 2, 3, 4, 6, 8, and 12 h), one subgroup of mice from each main group was anesthetized with Zoletil^®^ 50. Systemic blood was collected and heparinized to prepare the plasma. Next, the animals were immediately euthanized by cervical dislocation under deep anesthesia prior to liver and lung excision. The excised liver and lungs were rinsed with ice-cold water and homogenized in ice-cold water. All samples were frozen at −80 °C until DTX concentration analysis was performed using a previously described LC-MS/MS method.

### 4.8. Pharmacokinetics of DiR-Labelled LZ-Nnps-DTX

#### 4.8.1. Preparation of DiR Labelled LZ-Nnps-DTX

Lyophilized LZ-Nnps powder was dissolved in water and heated at 70 °C for 30 min. Undissolved material was removed by centrifugation at 10,000 rpm for 10 min. The supernatant was collected, and the protein concentration was determined using a BCA kit (Beyotime Biotechnology, Shanghai, China) and adjusted to 1 mg/mL.

An ethanol solution containing DTX and the fluorescent dye DiR (mass ratio = 9:1) was slowly added dropwise to an aqueous solution of the LZ-Nnps. The final ethanol concentration was maintained below 1%, and the mass ratio of DTX to LZ-Nnps was adjusted to 0.3:1. The mixture was continuously stirred for 2 h. The pH of the solution was adjusted to 10.0 using NaOH or HCl solution, followed by continuous stirring for 12 h.

The mixture was centrifuged at 10,000 rpm for 10 min, and the supernatant was collected. It was then dialyzed for 24 h using a dialysis bag (molecular weight cut-off: 10,000 Da) and lyophilized to obtain DiR-labelled LZ-Nnps-DTX nanoparticles (hereafter referred to as LZ-Nnps-DTX-DiR).

For characterization, appropriate amounts of DiR dye, LZ-Nnps, LZ-Nnps-DTX, and LZ-Nnps-DTX-DiR powders were weighed. DiR was dissolved in ethanol and then diluted with water, and the other powders were dissolved directly in water to prepare aqueous solutions. A full ultraviolet–visible scan was performed over a range of 200–800 nm. The success of DiR labeling was determined based on the changes in the absorption spectra.

#### 4.8.2. Uptake of LZ-Nnps-DTX-DiR by Caco-2 Cells

Caco-2 human colorectal adenocarcinoma cells were obtained from the National Collection of Authenticated Cell Cultures (Shanghai, China). The cells were cultured at 37 °C in a humidified 5% CO in DMEM. Caco-2 cells (5 × 10^5^ cells/well) were seeded in 6-well plates and cultured for 24 h. Cells were then washed with Hank’s Balanced Salt Solution (HBSS) and divided into 6 groups: 2 control groups (maintained in fresh HBSS) and 4 inhibitor-treated groups. The inhibitor-treated groups were incubated with one of the following endocytosis inhibitors for 1 h: caveolae-mediated endocytosis inhibitor indomethacin (100 μg/mL), clathrin-mediated endocytosis inhibitor chlorpromazine (10 μg/mL), macropinocytosis inhibitor amiloride (2.5 mM), or phagocytosis inhibitor cytochalasin D (5 μM) [7]. After inhibitor pre-treatment, the cells were washed and incubated with either fresh HBSS (control) or LZ-Nnps-DTX-DiR (100 μg/mL) for 2 h. Cells were washed with ice-cold PBS, harvested, and resuspended in PBS. A total of 1 × 10^4^ cells per sample were analyzed using a Beckman CytoFLEX LX flow cytometer (Beckman Coulter Life Sciences, Indianapolis, IN, USA) with the APC-A750 channel (excitation laser: 650 nm; bandpass filter: 750 nm).

#### 4.8.3. Live Imaging Analysis in Mice

SPF male Kunming mice (22–24 g) were fasted for 12 h, with free access to water. Mice were orally administered an aqueous solution of LZ-Nnps-DTX-DiR at a dose of 100 mg/kg. After anesthesia, the mice were placed in an IVIS SPECTRUM Small Animal Live Imaging System (PerkinElmer, Waltham, MA, USA) for live imaging at 40, 120, 240, and 360 min after administration. Live imaging scans were performed using the epi-illumination mode with DiR excitation and emission wavelengths of 748 and 780 nm, respectively.

### 4.9. In Vitro Bioactivity of LZ-Nnps-DTX and LZ-Nnps

#### 4.9.1. Cytotoxicity of LZ-Nnps-DTX on Tumor Cells

The human cancer cell lines HepG2 (liver carcinoma, RRID:CVCL_0027), A549 (non-small cell lung cancer, RRID:CVCL_0023), and HCT116 (colon cancer, RRID:CVCL_0291) were obtained from the National Collection of Authenticated Cell Cultures (Shanghai, China). Cells were cultured at 37 °C in a humidified 5% CO_2_ atmosphere: HepG2 and A549 in DMEM, and HCT116 in RPMI 1640 medium. All culture medium was supplemented with heat-inactivated fetal bovine serum FBS (10%, *v*/*v*), penicillin (100 U/mL), streptomycin sulfate (100 μg/mL), 4-(2-hydroxyethyl)-1-piperazineethanesulfonic acid (HEPES; 15 mM), and sodium pyruvate (1 mM).

DTX was dissolved in DMSO to a stock concentration of 20 mg/mL and then diluted with culture medium to final concentrations of 0.3, 1, 3, 10, 30, and 100 ng/mL. Sterilized LZ-Nnps-DTX was directly dispersed in the culture medium to prepare serial solutions of the same final DTX concentrations (0.3–100 ng/mL). Sterilized LZ-Nnps were serially dispersed in the culture medium to match the LZ-Nnps concentrations present in the LZ-Nnps-DTX solutions. The final concentrations of FBS and DMSO in all the solutions were standardized to 2.5% and 0.5%, respectively.

Cells were seeded in 96-well plates and incubated with DTX, LZ-Nnps, or LZ-Nnps-DTX solutions for 48 h. After incubation, 10 µL of enhanced CCK-8 solution was added to each well. Following a 1 h incubation, the absorbance of each well at 490 nm was measured using a Thermo Fisher Multiskan SkyHigh microplate spectrophotometer (Thermo Fisher Scientific, Waltham, MA, USA) to assess cell viability.

#### 4.9.2. Uptake of LZ-Nnps-DTX-DiR by Raw264.7 Cells

The protocol for this assay was identical to that described in “Uptake of LZ-Nnps-DTX-DiR by Caco-2 Cells,” except that the incubation time was 1 h.

#### 4.9.3. Effect of LZ-Nnps Pretreatment on Macrophage Antitumor Activity

RAW264.7 cells were seeded in 6-well plates at a density of 5 × 10^5^ cells/well. After 24 h, the medium was replaced with a fresh medium. The LZ-Nnps powder was dispersed in a culture medium supplemented with 2.5% FBS to an initial concentration of 1 mg/mL and then diluted to final concentrations of 0.1, 0.3, and 1 μg/mL. RAW264.7 cells were treated with this series of LZ-Nnps concentrations. After an additional 24 h incubation, the supernatant was collected and transferred to HepG2 and HCT116 cells seeded in 96-well plates at 8 × 10^3^ cells/well and incubated for 24 h. Finally, the viability of HepG2 and HCT116 cells was assessed using the CCK-8 kit.

#### 4.9.4. Effects of LZ-Nnps on NO Release in Macrophages

RAW264.7 murine macrophages were seeded in 96-well plates at 5 × 10^4^ cells/well and treated with LZ-Nnps at serial concentrations of 0.3, 1, and 10 μg/mL. After 24 h of incubation, 100 μL of culture medium was mixed with an equal volume of Griess reagent [1% (*w*/*v*) sulfanilamide in 5% (*v*/*v*) phosphoric acid and 0.1% (*w*/*v*) N-(1-naphthalene) ethylenediamine dihydrochloride] and incubated at room temperature for 20 min. The absorbance at 550 nm was measured using a microplate reader to quantify NO. The levels of NO were normalized to the cell viability detected using a CCK-8 kit.

#### 4.9.5. Effects of LZ-Nnps on TNF-α Release in Macrophages

RAW264.7 cells were randomly seeded in 6-well plates at a density of 1 × 10^6^ cells/well and cultured for 24 h. The cells were then incubated with LZ-Nnps-DTX at 0.1, 0.3, or 1 μg/mL for 24 h. Supernatants were collected, and TNF-α levels were measured according to the manufacturer’s instructions for the TNF-α detection kit. The levels of TNF-α were normalized to the cell viability detected using a CCK-8 kit.

### 4.10. Data Analysis

Quantitative data are expressed as the mean ± SD. The median inhibitory concentration (IC_50_) and half-maximal effective concentration (EC_50_) values for DTX, LZ-Nnps, or LZ-Nnps-DTX were calculated using GraphPad Prism software (Version 9). Non-compartmental analysis was performed using the WinNonlin^®^ software (Version 6, Pharsight, CA, USA) to calculate the pharmacokinetic parameters of DTX.

For the dissolution and in vitro absorption experiments, the difference between the DTX and LZ-Nnps-DTX treatment groups as a whole was determined based on a two-way analysis of variance, while the difference between the two treatments at a certain time point was determined using Student’s *t*-test.

A *p*-value less than 0.05 was considered significant, and a *p*-value less than 0.01 was considered a very significant difference.

## 5. Conclusions

For the first time, protein-dominant natural nanoparticles, i.e., LZ-Nnps, were isolated from Ganoderma lucidum aqueous extract. Using a heat-induced self-assembly method, we achieved near-complete DTX encapsulation (EE = 99.97 ± 0.02%) and converted DTX from crystalline to amorphous state, enhancing its aqueous solubility by 11.4-fold and dissolution in GI fluids by 2.5–3.2-fold. Oral administration of LZ-Nnps-DTX in mice increased plasma C_max_ by about 34-fold (75.7 vs. 2.2 ng/mL) and AUC_0-t_ by about 7.8-fold (41.3 vs. 5.3 ng·h/mL) relative to free DTX, with markedly enhanced distribution to liver (C_max_ 203.5 vs. 27.8 ng/g) and lung (C_max_ 86.2 vs. 33.8 ng/g). Cytotoxicity against HepG2, A549, and HCT116 cells was largely retained. Critically, LZ-Nnps-DTX activated RAW264.7 macrophages to release TNF-α and NO, exerting synergistic antitumor immunity. In summary, LZ-Nnps-DTX represents a naturally derived, organic-solvent-free platform that achieves near-complete drug encapsulation, substantially improves pharmacokinetic profiles, and intrinsically activates macrophages, thereby offering a dual chemo-immunotherapeutic advantage not typically associated with conventional synthetic carriers. These findings support the potential of LZ-Nnps-DTX as an oral delivery system for DTX with synergistic antitumor benefits.

## Figures and Tables

**Table 1:** Pharmacokinetic parameters of DTX in the systemic circulation, livers, and lungs of the mice after oral administration of 20 mg/kg DTX or LZ-Nnps-DTX with an equivalent dose of DTX (mean, n = 5).

Samples	Parameters	DTX	LZ-Nnps-DTX
Systemic circulation	T_max_ (h)	2.0	0.083
	C_max_ (ng/mL)	2.2	75.7
	t_1/2_ (h)	3.1	1.6
	AUC_0-t_ (ng·h/mL)	5.3	41.3
	MRT (h)	2.7	1.4
Liver	T_max_ (h)	3.0	0.083
	C_max_ (ng/g liver)	27.8	203.5
	t_1/2_ (h)	1.2	2.2
	AUC_0-t_ (ng·h/g liver)	109.7	356.3
	MRT (h)	3.2	2.3
Lung	T_max_ (h)	0.083	0.083
	C_max_ (ng/g lung)	33.8	86.2
	t_1/2_ (h)	2.6	48.4
	AUC_0-t_ (ng·h/g lung)	167.9	528.0
	MRT (h)	5.4	5.2

AUC, area under the concentration-time curve; C_max_, peak concentration; MRT, mean retention time; t_1/2_, elimination half-life; T_max_, time to reach peak concentration.

## Data Availability

The original contributions presented in this study are included in the article/Supplementary Material. Further inquiries can be directed to the corresponding authors.

## Supplementary Materials

The following supporting information can be downloaded at: https://www.mdpi.com/article/10.3390/ph19060899/s1, Table S1. Results of the orthogonal experiments (mean ± SD, n = 3); Figure S1. The zeta potential of LZ-Nnps in water solutions with different pH values (mean ± SD, n = 3); Figure S2. UV-Vis absorption spectrum of DiR-labeled LZ-Nnps-DTX.

## Abbreviations

The following abbreviations are used in this manuscript.

AUC	Area under the concentration–time curve
BCA	Diquinolinic acid
C_max_	Peak concentration
DDS	Drug delivery systems
DiR	1,1′-dioctadecyl-3,3,3′,3′-tetramethylindotricarbocyanine iodide
DLS	Dynamic light scattering
DMEM	Dulbecco’s modified Eagle’s medium
DMSO	Dimethyl sulfoxide
DSC	Differential scanning calorimetry
DTX	Docetaxel
EC_50_	Half-maximal effective concentration
FBS	Fetal bovine serum
FTIR	Fourier transform infrared spectroscopy
HBSS	Hank’s balanced salt solution
HEPES	(4-(2-hydroxyethyl)-1-piperazineethanesulfonic acid
IC_50_	Median inhibitory concentration
IS	Internal standard
LC-MS/MS	Liquid chromatography tandem mass spectrometry
LZ-Nnps	Ganoderma natural nanoparticles
LZ-Nnps-DTX	Ganoderma natural nanoparticles loaded with docetaxel
MRT	Mean retention time
Nnps	Natural nanoparticles
NO	Nitric oxide
OD	Optical density
PDI	Polydispersion index
PXRD	Powder X-ray diffraction
RSA	Radical scavenging activity
SEM	Scanning electron microscope
SGFP	Simulated gastric fluid containing pepsin
SIFT	Simulated intestinal fluid containing trypsin
SPF	Specific-pathogen-free
t_1/2_	Half-life
TCM	Traditional Chinese medicine
Tmax	Time to reach peak concentration
TNF-α	Tumor necrosis factor-α
UPLC	Ultra-high performance liquid chromatography
