# Supplementary material for: Docetaxel Oral Delivery System Using Natural Nanoparticles Derived from Ganoderma: Enhanced Pharmacokinetics, Potent Cytotoxicity, and Macrophage-Activating Properties
Source: Pharmaceuticals (Basel). 2026 Jun 5;19(6):899. doi: 10.3390/ph19060899 (PMC13304593; doi:10.3390/ph19060899)
Supplement: Supplementary file 1 [file pharmaceuticals-19-00899-s001.zip › pharmaceuticals-4290042-supplementary.pdf]

Supplementary materials

S1 Orthogonal-designed experiments for the preparation of LZ-Nnps-DTX

S1.1 Basic procedures

The orthogonal-designed experiments were conducted according to the procedures described below and replicated three times.

LZ-Nnps powder was dispersed in water at a concentration of about 5 mg/mL. Then, the water solution of LZ-Nnps was heated to 65, 70, or 75°C for 0.5 hours. After that, the undispersed part was removed by centrifugation (10,000 rpm for 10 min). The content of Ganoderma protein in the resulting supernatant was measured by BCA analysis. The supernatant was then diluted to contain 0.6, 0.8, or 1 mg/mL of Ganoderma protein. Next, the ethanol solution of DTX was added to the LZ-Nnps solution while stirring (500 rpm), and the final concentration of DTX was 0.2, 0.3, or 0.4 mg/mL, and the final concentration of ethanol was kept below 1%. After that, the pH of the solution was adjusted to 8, 9, or 10, and the solution was continued to be stirred at 500 rpm for 12 hours. The solution was centrifuged at 10,000 rpm for 10 minutes and then lyophilized to obtain the powder of LZ-Nnps-DTX.

S1.2 Experiment design, results, and discussion

The orthogonal-designed experiments have four factors and three levels. The design and results of the experiments are shown in Table S1.

Table S1 Results of the orthogonal experiments (mean ± SD, n = 3).

Tests	Factors				Results		
	A	B	C	D	Size (nm)	Zeta potential (mV)	PDI
1	1	1	1	1	699.63 ± 357.04	-30.20 ± 9.19	0.71 ± 0.25
2	1	2	2	2	575.18 ± 117.24	-32.03 ± 3.96	0.63 ± 0.13
3	1	3	3	3	562.83 ± 124.45	-31.86 ± 6.35	0.61 ± 0.11
4	2	1	2	3	543.87 ± 263.12	-32.33 ± 7.29	0.64 ± 0.17
5	2	2	3	1	477.77 ± 103.74	-34.11 ± 3.34	0.57 ± 0.07
6	2	3	1	2	505.13 ± 38.82	-36.64 ± 2.67	0.61 ± 0.07
7	3	1	3	2	574.60 ± 112.32	-36.19 ± 4.46	0.66 ± 0.13
8	3	2	1	3	439.03 ± 123.80	-43.56 ± 5.06	0.52 ± 0.05
9	3	3	2	1	386.56 ± 103.33	-35.00 ± 6.65	0.56 ± 0.09

A, temperature (°C; 1, 2, 3: 65, 70, 75, respectively); B, LZ-Nnps concentration (mg/mL; 1, 2, 3: 0.6, 0.8, 1, respectively); C, DTX/LZ-Nnps ratio (1, 2, 3: 0.2, 0.3, 0.4, respectively); D, pH value (1, 2, 3: 8, 9, 10, respectively).

The variance analysis showed that (1) the primary factor influencing the size of LZ-Nnps-DTX was temperature ($p < 0.01$), followed by LZ-Nnps concentration ($p < 0.05$); (2) the key factor affecting the PDI of LZ-Nnps-DTX was temperature ($p < 0.05$), followed by LZ-Nnps concentration ($p < 0.05$); and (3) the predominant factor impacting the Zeta potential of LZ-Nnps-DTX was temperature ($p < 0.01$), followed by pH value of solution ($p < 0.05$).

Given that the PDI of LZ-Nnps-DTX nanoparticles exhibited minimal variation under different experimental conditions and the Zeta potential consistently remained significantly lower than -25 mV across all tested conditions, it is evident that particle size serves as the primary parameter for optimizing the preparation process.

Finally, in order to minimize the particle size, $A_2B_3C_2D_3$ was identified as the optimal preparation conditions for LZ-Nnps-DTX, with a heating temperature of 70°C, a protein concentration of 1 mg/mL, DTX/LZ-Nnps ratio of 0.3, and a solution pH value of 10.

S2 Zeta potential of LZ-Nnps

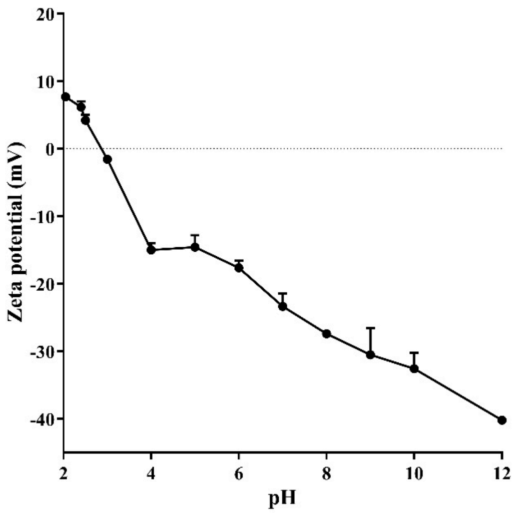

Figure S1 The zeta potential of LZ-Nnps in water solutions with different pH values (mean ± SD, n = 3).

S3 Labeling of LZ-Nnps-DTX with DiR

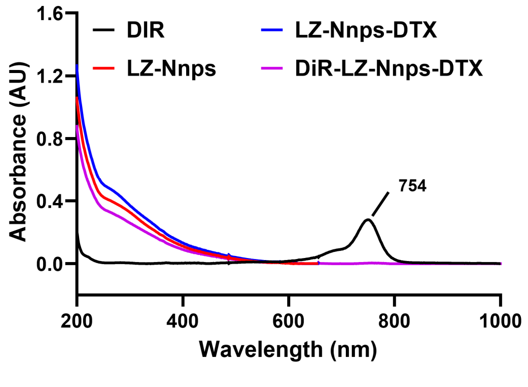

Figure S2 UV-Vis absorption spectrum of DiR-labeled LZ-Nnps-DTX.
